# Genomic Profiling of Thyroid Nodules: Current Role for ThyroSeq Next-Generation Sequencing on Clinical Decision-Making

**DOI:** 10.4274/2017.26.suppl.04

**Published:** 2017-01-09

**Authors:** Atil Y. Kargi, Marcela Perez Bustamante, Seza Gulec

**Affiliations:** 1 University of Miami Hospital, Clinic of Diabetes and Metabolism, Division of Endocrinology, Florida, USA; 2 Florida International University Herbert Wertheim College of Medicine, Departments of Surgery and Nuclear Medicine, Miami, USA

**Keywords:** thyroid cancer, Thyroid nodule, genomic profiling, next-generation sequencing, ThyroSeq, molecular testing

## Abstract

In recent years there has been an increased awareness of the genetic alterations underlying both benign and malignant neoplasms of the thyroid. Next-generation sequencing (NGS) is an emerging technology that allows for rapid detection of a large number of genetic mutations in thyroid fine-needle aspiration (FNA) specimens. NGS for targeted mutational analysis in thyroid tumors has been proposed as a tool to assist in the diagnosis of thyroid nodules with indeterminate FNA cytology. Results of genomic testing of thyroid nodules and thyroid cancers could also have prognostic implications and play a role in determining optimal treatment strategies including targeted therapies. We provide a critical review of existing studies assessing the performance of the ThyroSeq NGS test for the diagnosis and management of patients with thyroid nodules with indeterminate cytopathology and discuss the applicability of findings from these studies to clinical practice. While there are early indications to suggest a possible utility of data obtained from NGS to aid in prognostication and therapeutic decision-making in thyroid cancer, we recommend judicious use and cautious interpretation of such molecular testing until results of ongoing clinical trials become available. Lastly, we discuss recommendations provided from clinical practice guidelines regarding the use of mutation detection via NGS in the diagnostic evaluation of thyroid nodules.

## Abstract (tr)

Son yıllarda benign ve malign tiroid neoplazmlarının altında yatan genetik değişiklerle ilgili artan bir farkındalık oluşmuştur. Yeni nesil dizinleme [Next-generation sequencing (NGS)] tiroid ince iğne aspirasyon (İİA) örneklerinde çok sayıda genetik mutasyonun hızlı tespitine izin veren ve gelişmekte olan bir teknolojidir. Tiroid tümörlerinin hedeflenen mutasyon analizi için NGS, İİA ile belirsiz sitoloji saptanan tiroid nodüllerinin tanısında yardımcı olmak için bir araç olarak öne sürülmüştür. Ek olarak, tiroid nodülü ve tiroid kanserlerinin genomik test sonuçlarının prognostik etkileri olabilir ve hedefli tedaviler dahil olmak üzere uygun tedavi stratejilerinin belirlenmesinde önemli bir rol oynayabilir. Bu yazıda İİA ile belirsiz sitolojisi olan tiroid nodüllü hastaların tanı ve tedavisinde ThyroSeq NGS testinin performansını değerlendiren mevcut çalışmaların bir derlemesini sunmayı ve bu çalışmalardan elde edilen bulguların klinik pratikte uygulanabilirliğini tartışmayı amaçladık. Her ne kadar erken veriler NGS’den elde edilen verilerin tiroid kanserinde prognoz ve tedavi kararı sürecine olası yardımlarını öne sürse de, bu tür moleküler testlerin devam eden klinik çalışmaların sonuçları belirlenene kadar, akıllıca kullanılmasını ve dikkatli yorumlanmasını öneriyoruz. Son olarak, tiroid nodüllerinin tanısal değerlendirilmesinde NGS ile mutasyon tespiti ile ilgili klinik uygulama kılavuzlarından edinilmiş öneriler tartışılmıştır.

## INTRODUCTION

Thyroid nodules are common in the general population with higher prevalence in women and in older persons. When ultrasound is performed at random in the general population 19-68% of individuals are found to harbor one or more thyroid nodules (1). While the majority of these nodules are not clinically significant 7-15% are malignant (2). Paralleling the increased use of imaging techniques and of thyroid fine-needle aspiration (FNA) there has been dramatic increase worldwide in both the incidence of thyroid nodule diagnosis and that of thyroid cancer over the past 20-30 years (3,4).

Several clinical practice guidelines have set forth strategies to manage patients who are discovered to have thyroid nodules, yet a great deal of controversy still exists as to the optimal approach to diagnosis and treatment (5,6). The widespread use of high-resolution ultrasonography of the neck as well as thyroid FNA has significantly enhanced our ability to diagnose malignancy among thyroid nodules, however 20-30% of cytology results from thyroid FNA fall into one of three indeterminate diagnostic categories according to the Bethesda System for Reporting Thyroid Cytopathology: Atypia of undetermined significance/Follicular lesion of undetermined significance (AUS/FLUS) (Bethesda category III), follicular neoplasm/suspicious for follicular neoplasm (FN/SFN) (Bethesda category IV), and Suspicious for malignancy (SM) (Bethesda category V) (7). The reported frequency and risk of malignancy with each of the Bethesda reporting categories is summarized in Table 1.

Patients and physicians faced with an indeterminate cytopathology report will have to make the sometimes difficult decision of deciding on the next step in management of the thyroid nodule, which until recent years has meant choosing from one of three options: repeat FNA, observation with continued ultrasound surveillance or surgical management. Each of these strategies brings with it specific considerations and complexities; for instance in patients referred for surgery the need to decide upon the extent of thyroidectomy and the potential need for a two-step procedure of thyroid lobectomy followed by a completion thyroidectomy in the circumstance that the lobectomy results in a diagnosis of thyroid cancer.

Taking into consideration that many thyroid cancers are indolent tumors and that many patients may have an excellent prognosis even when the diagnosis and treatment has been delayed and the fact that most patients who undergo thyroidectomy for AUS/FLUS and FN/SFN cytopathology will be diagnosed with benign nodules on final surgical histopathology, clinicians and patients have been left with weighing the risks of a more conservative strategy of surveillance with that of the more aggressive approach of proceeding to thyroidectomy.

A variety of factors can predict the risk of cancer and aid in the decision on optimal management for patients presenting with nodules having indeterminate cytopathology; including patient risk factors (age, gender, family history, past exposure to ionizing radiation), serum TSH level and presence or absence of sonographic features suspicious for papillary thyroid cancer (PTC) (6,8,9). In their 2015 management guidelines pertaining to adults with thyroid nodules, the American Thyroid Association (ATA) has provided clear guidance on the criteria that should be used to determine the initial indication for FNA based on traditional risk factors and in particular a risk stratification model heavily reliant upon sonographic appearance of the nodule (6).

However, in the circumstance that FNA is performed, once patients and health care providers are faced with indeterminate cytology, it becomes much less clear from the guidelines precisely how the same criteria should be used to inform management decisions. This situation has created a need to improve on the cytological inaccuracy inherent to the diagnosis of indeterminate thyroid FNA, resulting in the development of a number of new diagnostic modalities intended for application as a “rule-in” or “rule-out” test for thyroid cancer. When discussing the performance of any of these tests it must be taken into consideration that the ideal ‘rule-in’ test should have a positive predictive value (PPV) similar to that of a malignant cytological diagnosis (Bethesda category VI) (98.6%), while an ideal ‘rule-out’ test should have a negative predictive value (NPV) comparable to that of benign cytology (Bethesda category II) (96.3%) (7).

In recent years a number of diagnostic tests have been evaluated to aid in the diagnosis of indeterminate thyroid nodules, including FDG-PET and several assessments of molecular markers in FNA specimens (10,11). Molecular tests include immunohistochemistry for Galectin -3, HBME-1 and CK19; gene expression and microarray analysis; microRNA expression; and testing for mutations and gene rearrangements (6,12). Currently in the U.S.A. commercially available molecular tests include those for single or multiple mutation analysis, combination panels for mutation analysis and chromosomal rearrangements (miRInform®-Asuragen, ThyroSeq-CBLPath and University of Pittsburgh Medical Center) and a proprietary gene expression classifier (Afirma GEC®-Veracyte) (13,14,15). While initially the gene expression classifier (GEC) was proposed as the best among these tests to rule-out malignancy and mutation analysis was preferred as a “rule-in”, the next-generation sequencer (NGS) ThyroSeq has recently been shown to have both a high PPV and NPV for thyroid cancer diagnosis when applied to thyroid FNA with indeterminate cytology (16,17,18). A further strength of the ThyroSeq, when compared to GEC, is that it provides detailed and specific information regarding the exact genetic alteration driving the disease, which could potentially provide prognostic and therapeutic implications including impacting upon extent of surgery, use of RAI and possible future targeted therapies.

Thyroid cancer, like all cancers, is a disease of the genome. The initiation and progression of cancer is due to the accumulation of genetic and epigenetic changes such as somatic mutations, chromosomal rearrangements, micro RNA dysregulation and alterations in gene expression (19). In differentiated thyroid cancer (DTC), the observed genetic changes frequently lead to activation of the MAPK or PI3K-AKT pathways. Approximately 70% of DTC demonstrate one of four genetic abnormalities: point mutations in the BRAF or RAS gene or either one of two chromosomal rearrangements: RET/PTC or PAX8/PPARG (19). Our knowledge of the genomic alterations explaining the remaining approximately 30% of all thyroid cancers not harboring one of the aforementioned four genetic aberrations has been greatly expanded by a number of recent discoveries, including those reported in 2014 by the National Cancer Genome Atlas Research Network, in which the genetic driver was identified in 96.5% of 496 PTC cases (20,21). The findings of this last report have led some experts to propose a reclassification of PTCs based on molecular characteristics to better reflect their underlying differentiation and signaling properties (21). While a detailed discussion of all current molecular tests in thyroid FNA is beyond the scope of this article, our review will focus on the role of NGS, a methodology which we believe may hold particular promise in diagnosis of thyroid FNA as well as future potential for use in prognostication and informing management of patients with thyroid cancer.

## NEXT-GENERATION SEQUENCING IN THYROID FINE-NEEDLE ASPIRATION

NGS is a method of simultaneous sequencing of a very large number of short nucleic acid sequences that can be used to detect multiple genetic alterations in large regions of the genome (22). Compared to standard methods of sequencing, such as Sanger sequencing, NGS has the advantage of rapid simultaneous sequencing of large sections of the genome and quantitative assessment of mutated alleles. NGS can be used for whole-genome sequencing as well as in a more targeted manner directed at specific mutations in specific areas of the genome.

The ThyroSeq NGS panel provides simultaneous sequencing for detection in over a thousand hotspots of 14 thyroid cancer-related genes and for 42 types of gene fusions occurring in thyroid cancer (14,17). The genes analyzed for mutation are *AKT1, BRAF, CTTNB1, GNAS, HRAS, KRAS, NRAS, PIK3CA, PTEN, RET, TP53, TSHR, TERT* and *EIF1AX*. The gene list for gene fusions and expression consists of *RET, PPARG, NTRK1, NTRK3, ALK, IGF2BP3, BRAF, MET, CALCA, PTH, SLC5A5, TG, TTF1, KRT7* and *KRT20*.

The proposed uses for NGS for thyroid FNA samples include diagnosis of cytologically indeterminate thyroid nodules, prognostication in thyroid cancer and to inform selection of targeted therapies (14). The possible applications and indications of ThyroSeq include:

1. Thyroid FNA with indeterminate cytology (Bethesda categories III, IV and V),

2. Malignant thyroid cytology (Bethesda category VI), when results of the NGS are expected to affect the decision for extent of oncological surgery,

3. Benign thyroid cytology (Bethesda category II), when strong SM exists on clinical grounds such as presence of a highly suspicious sonographic pattern,

4. When the diagnosis of thyroid cancer is established cytologically or histologically and molecular profiling will effect decision regarding radioactive iodine therapy, intensity of follow up, or for selection of targeted therapies in patients with advanced cancer.

We will discuss the potential roles of NGS in thyroid FNA specimens below, with an emphasis on its role in clinical decision-making.

## POTENTIAL ROLE OF NEXT-GENERATION SEQUENCER IN AUS/FLUS (BETHESDA CATEGORY III) CYTOLOGY

The diagnosis of AUS/FLUS should be made in FNA specimens containing cells with architectural and/or nuclear atypia more pronounced than expected for benign changes, yet not sufficient to be classified in one of the higher risk Bethesda categories (7). Although this diagnosis has an expected and recommended frequency of 7%, recent analyses have found this cytological category to be diagnosed in 1-27% of all thyroid FNA specimens (23). In studies assessing the risk of cancer in patients with Bethesda category III nodules, the rate of malignancy diagnosed in patients who went to surgery was 6-48%, with a mean risk of 16% (24).

**Figure 1:**
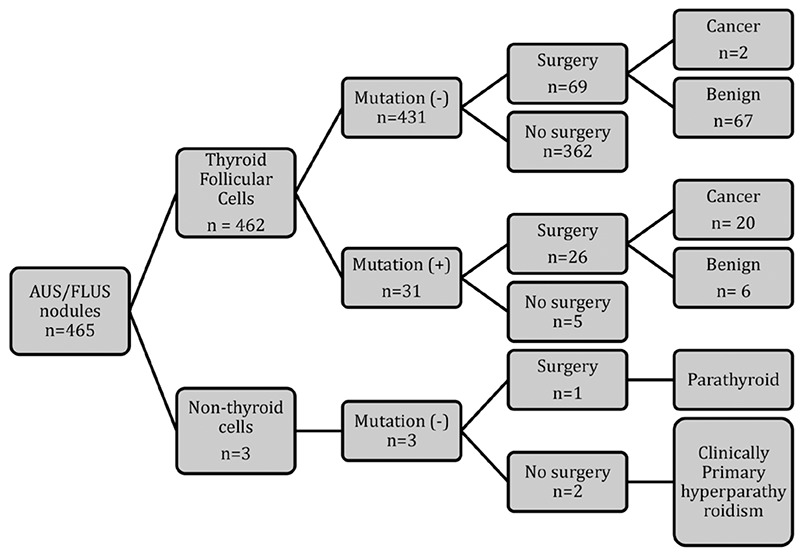
Schematic representation of study flow and overall performance of ThyroSeq in thyroid nodules with atypia of undetermined significance/follicular lesion of undetermined significance. Results showed sensitivity 90.0% [confidence interval (CI) 78.8-100], specificity 92.1% (CI 86.0-98.2), positive predictive value 76.9% (CI 60.7-93.1) and negative predictive value 97.2% (CI 78.8-100) with accuracy of 91.8% (CI 86.4-97.3). The overall prevalence of a thyroid cancer diagnosis in the study of all samples of follicular cells (n=462) that underwent molecular testing was 4.8%. (Adapted from Nikiforov YE, Carty SE, Chiosea SI, Coyne C, Duvvuri U, Ferris RL, Gooding WE, LeBeau SO, Ohori NP, Seethala RR, Tublin ME, Yip L, Nikiforova MN. Impact of the multi-gene ThyroSeq next-generation sequencing assay on cancer diagnosis in thyroid nodules with atypia of undetermined significance/follicular lesion of undetermined significance cytology. Thyroid 2015;25:1217-1223).

To date only one study has assessed the performance of ThyroSeq in AUS/FLUS (17). In this study 465 FNA samples from 441 patients at a single institution diagnosed as AUS/FLUS on cytology were submitted prospectively to ThyroSeq molecular testing. In addition to the 42 gene fusions and 14 genes analyzed for point mutation, expression of eight genes were analyzed to evaluate the cell composition of the needle aspirates. Ninenty-eight of the cases (21%) had a definitive diagnosis by either surgical (n=96) or non-surgical (n=2) methods. Of all FNA samples 462 were determined to be composed of follicular cells while three samples were diagnosed as parathyroid in origin. Among the samples consisting of follicular cells 31 were positive on mutational analysis (6.7%) (Figure 1).

Of the entire group of 441 patients, 96 nodules occurring in 90 patients were surgically removed due to the finding of an additional nodule in the same gland with either Bethesda V or Bethesda VI cytology in five patients. Twenty-seven patients underwent thyroidectomy because of positive ThyroSeq results and the remaining cases were reported by the authors to have been operated on based on patient preference. In all, 98 nodules from 92 patients had a definitive diagnosis, either surgical (n=96) or nonsurgical.

It is important to note that the study was conducted in a prospective manner, in that the molecular analysis was performed prior to the surgery. Therefore, the histopathologic diagnosis was provided by pathologists that were not blinded to results of the NGS test. Of all FNA samples deriving from follicular cells 31 (6.7%) were positive for mutations (n=24) or gene fusions (n=7). The most common genetic alteration encountered were mutations involving *RAS* (n=17) and only one nodule was found to be positive for the BRAFV600E mutation. Of the 31 nodules with positive ThyroSeq, 26 were surgically treated while 69 out of the total group of 431 mutation negative nodules, were subjected to surgical removal. Of the surgeries performed, half (n=45) were total thyroidectomies and the remaining half underwent hemi-thyroidectomy.

Among the 26 nodules with positive ThyroSeq results that underwent surgical treatment, 20 (77%) were ultimately deemed to be malignant by histopathology. Eighteen were follicular-variant papillary thyroid carcinoma and two represented the classic variant of papillary carcinoma. Of the six benign nodules that had tested positive for mutations, two had *NRAS* mutation and the others contained single mutations each in *HRAS, E1F1AX* or *PTEN* with one nodule harboring a *THADA* fusion. On histology, 4 out of the six benign nodules harboring mutations were classified as follicular adenomas and the other two were deemed to represent hyperplastic nodules.

Of the 69 thyroid nodules that were excised after testing negative by the next generation-gene sequencer only two were malignant on final pathology. Both tumors were papillary carcinomas, under two centimeters in diameter, confined to the thyroid and did not exhibit lymphovascular invasion.

On final analysis of test performance, ThyroSeq provided accurate classification of 91 out of 96 nodules in which a final surgical diagnosis was available as either benign (n=71) or malignant (n=20). Two false-negative and six false-positive tests were encountered in the study. Based on these findings the performance characteristics of the test were quite favorable with a 90.9% sensitivity, 92.1% specificity. The NPV was 97.2% and PPV 76.9%.

When interpreting the above performance characteristics of the ThyroSeq, it is important to note that while sensitivity and specificity are characteristics intrinsic to any test, the resulting PPV and NPV values are highly influenced by the pre-test probability of the disease, in other words the performance characteristics involving predictive value will change significantly based on the prevalence of disease in the study population. Because the prevalence of malignancy among AUS/FLUS that has been reported in the literature varies between 6% and 48%, the NPV of the molecular test would be expected to range from 99% to 92%, and the PPV between 42% and 91%.

Given the high sensitivity of the test for diagnosing thyroid cancer and the resultant NPV, which is similar to that reported for benign cytology (<5%), it has been proposed that a negative ThyroSeq in a patient with AUS/FLUS can generally be considered as a basis for observation rather than surgery (17). The exception could be a population or particular patient or nodule with a high pre-test probability for cancer.

Though the addition of several genetic markers to the previously reported seven-gene panel has resulted in a decrease of PPV from 88% to 77%, the PPV for the ThyroSeq may still be sufficient to consider it not only as a rule-out test, but also as a rule-in test for the diagnosis of thyroid cancer. A further strength of NGS is that the PPV is close to 100% in the case of certain mutations including in tumors positive for the most common BRAF mutations and for fusions in *PPARG, NTRK1, NTRK3* and *ALK*. One must also take into consideration that 3 out of the 6 total “false-positives” in this study were benign nodules harboring *RAS* mutations. These are clonal neoplasms and there is controversy that such tumors could represent pre-malignant lesions. In fact, several lines of evidence lend support to the hypothesis that RAS is an oncogene responsible for gradual progression from benign to malignant thyroid lesions (25).

Though the above described findings are encouraging, there are several limitations of the study. The study was performed at a single institution and the participants, including the patients, clinicians, surgeons and pathologists were not blinded to the results of the molecular test. In fact, the results of the test were reported to have been used as a basis to operate in at least 27 of the cases. The unblinded nature of the study could lead to an overestimation of the test accuracy, a phenomenon known as review bias or expectation bias. Given the very short-term follow-up provided and the lack of surgical definitive diagnosis for the large number of cases that had negative mutation analysis and were not operated on, we cannot know for sure the performance characteristics of the test in the entire group of patients tested, which consists mainly of patients who did not undergo surgery and of which none had long-term follow-up at the time of reporting of the study findings. Of the 462 nodules of follicular cell composition that were submitted for molecular testing only 22 were ultimately diagnosed as malignant. Given the lack of long term follow up, it is reasonable to question whether some cases of thyroid cancer remained undiagnosed among the 367 nodules that were not surgically treated.

While the authors provided data regarding the rationale to proceed to surgery in the group of patients that were submitted to thyroidectomy, details regarding the decision-making process leading to observation in the cohort not operated on and therefore not included in the analysis of test performance could also be of use in understanding the full implications and the generalizability of the study results. What were the sonographic features of the nodules in the study? How did they correlate with results of mutation analysis and were they used in the decision making process to select nodules for surgery and on the extent of surgeries performed? What were the baseline characteristics of the patients and were other molecular tests such as GEC also performed and utilized in the decision to observe vs. proceed to surgery? The answers to these questions would be helpful in understanding how the results of the study could inform every day clinical practice.

Furthermore, while at first glance it appears to be a strength of this study that the majority of the patients who underwent thyroidectomy did so based on suspicious or malignant results of a co-existing nodule other than the nodule sampled and included in the analysis for test performance, this also may decrease the applicability of the test performance to the more common scenario in which patients undergo thyroidectomy for diagnosis of an indeterminate solitary nodule, without a co-existing nodule with a higher risk cytological diagnosis. Thyroid cancer, PTC in particular, is often multi-focal and patients harboring one malignant thyroid nodule may be more likely to have another. Whether the test would perform as well in a large cohort of patients with solitary nodules or co-existing benign nodules is a matter that demands further investigation.

To determine the true value of the NGS in clinical decision-making in this study population it would have also been helpful to know the sonographic and other traditional thyroid cancer risk factors of all the patients who had ThyroSeq testing. It is possible that in a significant number of these cases the pre-test probability of cancer may have been high (or low) enough to justify surgery or observation as the best management strategy, based on for instance very high (or low) risk sonographic nodule appearance. Also, it is possible that excluding patients who proceeded to surgery based on the mutation analysis results or due to “patient preference” from the calculations of test performance characteristics would have yielded different results.

Multi-center studies of ThyroSeq in which practitioners and participants are blinded to test results, with long-term follow-up including health outcomes data will provide even more value in assessing the performance of the ThyroSeq and its applicability and utility for “real-world” management of thyroid nodules with Bethesda III cytology. However, given the already recognized implications of the mutation analysis on diagnosis as well as emerging data suggesting its use in determining prognosis or selecting among treatment options in some cases, it may not be considered ethical, even at this early stage of inquiry into the role of molecular testing in diagnosing thyroid carcinoma, to withhold results of molecular testing from subjects enrolled in such studies.

## COMPARISON OF UTILITY OF NEXT-GENERATION SEQUENCER TO OTHER STRATEGIES RECOMMENDED FOR THE MANAGEMENT OF AUS/FLUS CYTOLOGY

In regards to the strategy aimed at diagnosing and managing patients with a Bethesda category III thyroid nodule cytology result, the 2015 ATA guidelines provide the following recommendations (6):

“For nodules with AUS/FLUS cytology, after consideration of worrisome clinical and sonographic features, investigations such as repeat FNA or molecular testing may be used to supplement malignancy risk assessment in lieu of proceeding directly with a strategy of either surveillance or diagnostic surgery. Informed patient preference and feasibility should be considered in clinical decision-making (weak recommendation, moderate-quality evidence). If repeat FNA cytology and/or molecular testing are not performed or inconclusive, either surveillance or diagnostic surgical excision may be performed for an AUS/FLUS thyroid nodule, depending on clinical risk factors, sonographic pattern, and patient preference (strong recommendation, Low-quality evidence).”

Prior to the availability of molecular testing for FNAs with AUS/FLUS cytology, it was recommended to consider repeat FNA as one approach to management (26). This was based on the observation that approximately 50% of such repeat FNAs resulted in benign cytology. However, a recent report has described similar rates of malignancy in patients undergoing surgery after benign results on repeat FNA and those with repeatedly Bethesda category III cytology (27). For those patients not wanting to be subjected to a repeat FNA procedure a second-opinion review of the original FNA specimen by a high-volume cytopathologist may result in reclassification and could be a reasonable first-step in some instances (28).

Ultrasound features of the nodule with AUS/FLUS cytology may be used to aid in improving diagnostic prediction of malignity or benignity (29,30). Retrospective studies have reported a PPV of 60-100% when suspicious sonographic appearance is present. However, these studies are limited by the fact that surgical diagnosis was not available for the majority of nodules and follow-up was short-term in duration. The combination of sonographic characteristics and molecular testing in AUS/FLUS has only been reported in one study using a GEC and none using mutational analysis or NGS (31). While this study did not show any benefit in improving prediction provided by the molecular testing alone, it may have not been adequately powered.

Though not commonly performed or recommended in the evaluation of thyroid nodules, fludeoxyglucose-positron emission tomography (FDG-PET) has been reported to have a high NPV when applied to the diagnosis of cytologically indeterminate thyroid nodules. In a systematic review and meta-analysis of 6 studies FDG-PET had a low PPV (39%) and a high NPV (96%) when performed in thyroid nodules with Bethesda category III or IV cytology (10).

While the optimal approach to the diagnosis and management of thyroid nodules with AUS/FLUS cytopathology remains controversial, molecular tests including NGS have been increasingly utilized to provide additional information to aid in the decision. In its 2015 guidelines the ATA conclude “Further research is needed to consider the impact of considering clinical and sonographic features on the potential utility and interpretation of molecular testing of FNA specimens.”

## CLINICAL UTILITY OF NEXT-GENERATION SEQUENCING IN FOLLICULAR NEOPLASM/SUSPICIOUS FOR FOLLICULAR NEOPLASM (BETHESDA CATEGORY IV) CYTOLOGY

According to the Bethesda system, the diagnosis of FN/SFN should be made in thyroid aspirates that have follicular cells arranged in an architectural pattern characterized by cell crowding and/or microfollicle formation and lacking nuclear features of papillary carcinoma, or are comprised almost exclusively of oncocytic (Hurthle) cells (7). These cytological patterns are seen with follicular and Hurthle cell carcinomas and the follicular variant of papillary carcinoma, however they are commonly observed in follicular adenomas and in hyperplastic nodules as well. Since such benign lesions are fairly common, they have a high false-positive rate on FN/SFN cytology, because only 14-34% of all nodules undergoing FNA with FN/SFN cytology are identified as malignant on the gold-standard surgical pathology (24).

In a meta-analysis of 8 studies with a total of 25,445 thyroid FNA samples, 10.1% of the results were reported as Bethesda IV with an average 26% rate of thyroid cancer diagnosed among these after surgery (32). The typical management approach has been to perform diagnostic lobectomy for such patients.

Prior to the recent introduction of ThyroSeq, available molecular tests improved either the PPV or the NPV for FN/SFN nodules, but not both at the same time. The GEC test, Afirma (Veracyte, South San Francisco, California), offers a high NPV, but its PPV is as low as 15% to 37% when applied to FN/SFN (33,34). As a result, the GEC may not be ideal to use as a basis to avoid diagnostic lobectomy in the majority of patients with this cytological diagnosis when classified as GEC suspicious, yet ultimately are found to have benign histology. The previously reported 7-oncogene panel yields a high PPV but a low NPV, which can aid in selecting patients with a higher risk of cancer and may help the surgeon decide on the appropriate extent of surgery, but does not prevent diagnostic surgeries for the majority of patients, in which the nodules are eventually determined to be benign (35). The low NPV of the 7-gene panel is due to the fact that only approximately 70% of thyroid cancer harbor a mutation in any of the 7 genes tested.

In the largest study of molecular marker testing in FN/SFN to date, Nikiforov and colleagues (16) reported findings of NGS (ThyroSeq) in 143 patients with FN/SFN cytology all of who underwent surgery for definitive diagnosis. The study included both a cohort of 91 patients in whom the molecular testing was performed retrospectively after surgery and final histopathologic diagnosis, as well as a cohort of 52 consecutive FNA samples studied prospectively in which the NGS was performed prior to thyroidectomy. While the researchers performing the molecular testing were blinded to the results of the surgical pathology, the pathologists reporting on the surgical specimens were not blinded entirely at the time of their analysis of the specimens. The ThyroSeq included testing for 13 mutant genes as well as 42 gene fusions known to occur in thyroid cancer. Expression of 8 other genes was assessed to confirm the cellular composition of the FNA sample.

**Figure 2:**
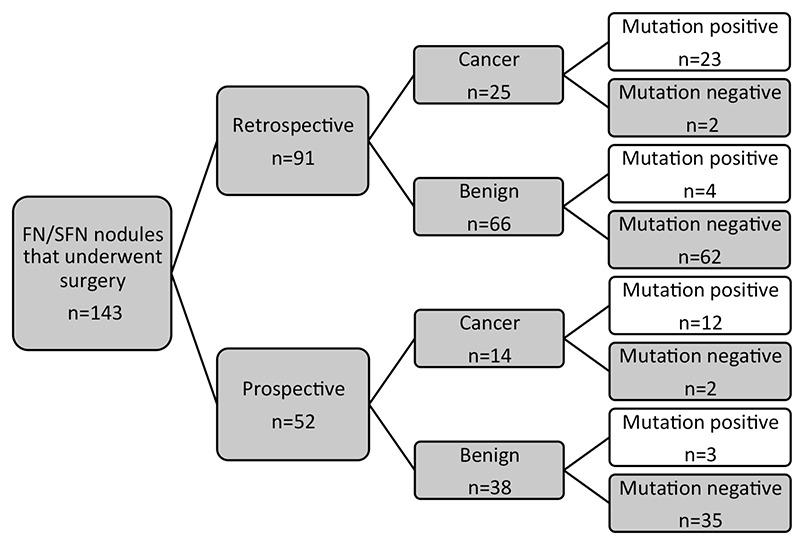
Schematic representation of study flow and overall performance of ThyroSeq in thyroid nodules with follicular neoplasm/suspicious for follicular neoplasm. Results showed sensitivity 90% [confidence interval (CI) 80-99], specificity 93% (CI 88-98%), positive predictive value 83% (CI 72-95) and negative predictive value 96% (CI 92-95) with accuracy of 92% (CI 88-97). (Adapted from Nikiforov YE, Carty SE, Chiosea SI, Coyne C, Duvvuri U, Ferris RL, Gooding WE, Hodak SP, LeBeau SO, Ohori NP, Seethala RR, Tublin ME, Yip L, Nikiforova MN. Highly accurate diagnosis of cancer in thyroid nodules with follicular neoplasm/suspicious for a follicular neoplasm cytology by ThyroSeq v2 next-generation sequencing assay. Cancer 2014;120:3627-3634). FN/SFN: Follicular neoplasm/suspicious for follicular neoplasm

Among the retrospective cohort (n=91) surgical pathology reporting was consistent with 66 benign nodules (35 follicular adenomas and 31 hyperplastic nodules) and 25 malignant nodules (Figure 2). The malignancy rate in this cohort was 27.5% with 3 FTC and 24 PTC, of which 19 were follicular variant PTC. The rate of malignancy was similar in the prospective cohort at 26.9% with 38 benign lesions and 14 malignant lesions, including 11 PTC and 3 FTC. As expected, a proportion of nodules were found to represent Hurthle cell tumors, the frequency of which was reported in detail for all groups.

The most frequent mutations identified were that of NRAS (n=16) and KRAS (n=6) in which the rate of cancer diagnosed on final histology was 81% and 83% respectively. HRAS mutation was discovered in two samples, both of which were malignant on final analysis. Only 1 out of 3 samples harboring a TSH-receptor gene mutation (TSHR) was malignant. Several other mutations, though encountered less frequently in the cohorts, had a much higher rate of malignancy of 100% including 4 out of 4 samples harboring TERT mutations and one each in samples with mutations in BRAF, TP53 and PI3K. All of the samples identified with gene fusions (n=9), were malignant, and involved one of the three genes PPARG, THADA and NTRK3.

Analysis of the data revealed no differences in operating characteristics among the 2 cohorts; therefore, they were combined to assess test performance. In the entire cohort of 143 patients, the test performed at a 90% sensitivity (95% confidence interval (CI), 80%-99%), 93% specificity (95% CI, 88%-98%), an NPV of 96% (95% CI, 92%-100%), and a PPV of 83% (95% CI, 72%-95).

Because NPV and PPV are greatly affected by prevalence of disease in the test population, the variable rates of malignancy for FN/SFN cytology at different institutions would be expected to alter predictive values of any test. In the review of 8 studies performed by Bongiovanni and colleagues (32), the cancer rate among Bethesda IV varied between 14% and 34% which would result in the ThyroSeq having a NPV between 98% and 95%, and PPV range between 68% and 87%.

Of note, of the four false-negative results representing thyroid malignancies without detected genetic abnormality, all four were intra-thyroidal and none had aggressive histopathological features. The authors speculated that the fact that no aggressive tumors were missed could be due to the fact that such tumors often have mutations in TERT, BRAF or more than one mutation. Three cancers in the series were reported to have more than one mutation. A recent case report further underscores the possible implications of the detection of multiple mutations and proposes a relationship of such a finding to aggressive tumor behavior (36).

Based on the above reported data it could be concluded that most patients with thyroid nodules with Bethesda IV cytopathology and negative NGS testing could be monitored without surgery. Notable exceptions may be in settings where the patient population has an unusually high prevalence of thyroid cancer or in individual patients in which the pre-test probability of cancer is exceptionally high due to other predictive factors such as family history, prior irradiation or high-risk sonographic characteristics of the nodule. In those unusual clinics having a high prevalence of thyroid cancer above 50% among their FN/SFN patient population, indeed the NPV of ThyroSeq would be below 90% and this could be considered too low to avoid diagnostic lobectomy.

The 2015 ATA guideline pertaining to the management of the patient with a thyroid nodule and FN/SFN cytology recommends:

“A) Diagnostic surgical excision is the long-established standard of care for the management of FN/SFN cytology nodules. However, after consideration of clinical and sonographic features, molecular testing may be used to supplement malignancy risk assessment data, in lieu of proceeding directly with surgery. Informed patient preference and feasibility should be considered in clinical decision-making (weak recommendation, moderate-quality evidence).

B) If molecular testing is either not performed or inconclusive, surgical excision may be considered for removal and definitive diagnosis of an FN/SFN thyroid nodule (strong recommendation, low-quality evidence)”.

## RECOMMENDATIONS ON THE USE OF NEXT-GENERATION SEQUENCING FROM CLINICAL PRACTICE GUIDELINES

In 2015 the ATA published revised guidelines on the management of adult patients with thyroid nodules and thyroid cancer (6). These guidelines included a thoughtful and detailed discussion of the potential role of molecular testing in the diagnosis of thyroid nodules and their implications for the management of patients with thyroid nodules and cancer. It should be noted that though the guideline included discussion of the previously reported 7 gene mutational analysis panel and the published reports of ThyroSeq in FN/SFN cytology, the more recent publication regarding performance of ThyroSeq in AUS/FLUS was not available at the time of publication of the 2015 ATA guidelines.

The authors of the ATA guidelines created a framework for the use of molecular testing including a classification of such tests according to intended use as either for diagnostic purposes (for classification of a disease state), prognostic or predictive purposes (to provide predictive information regarding the probability of benefit or harm of a specific treatment) (37). The ATA authors emphasize that since there is a lack of long-term outcomes data regarding the use of molecular testing to guide therapeutic decision-making, it remains unknown whether routine use of such tests in clinical practice would result in a net benefit in the health of patients with thyroid nodules and thyroid cancer. Similarly, a National Comprehensive Cancer Network task force report has declared that the clinical utility of any molecular test must be based on strong evidence that use of the test “improves patient outcomes sufficiently to justify its incorporation into routine clinical practice” (37).

Taking into account the above principles, the ATA guidelines recommend that the current use of molecular marker testing for patients with indeterminate thyroid nodule cytology is primarily diagnostic (to rule in or rule out malignancy), with an added implication of a companion use to aid in decision-making on initial surgical management (the decision to perform surgery and to guide the extent of surgery).

The 2015 ATA guidelines also point out that while previously published guidelines, including the ATA statement on surgical application of molecular profiling of thyroid nodules, were written at a time when total or near-total thyroidectomy was recommended as the initial surgical procedure for most cases of thyroid cancer, the current guidelines suggest more conservative surgical management (i.e., hemi-thyroidectomy) be considered as an option for low-risk thyroid cancer (38). This change could affect the utility of the result of NGS to determine extent of surgery for patients with indeterminate cytology and positive mutational analysis. Furthermore, there are no long-term outcome data testing the strategy of using NGS or other molecular tests in indeterminate FNA specimens to stratify surgical approach.

In summary the 2015 guidelines of the ATA provide the following recommendation: “If molecular testing is being considered, patients should be counseled regarding the potential benefits and limitations of testing, and about the possible uncertainties in the therapeutic and long-term clinical implications of results”.

## POTENTIAL USE OF MUTATIONAL ANALYSIS FOR PROGNOSTICATION AND TARGETED THERAPY OF THYROID CANCER

A strength of NGS when applied as a diagnostic test to patients with thyroid nodules and thyroid cancers is the potential impact that knowledge of the underlying genetic anomaly could have on prognostication and implications for treatment decisions. Mutations involving *AKT1, TP53, PIK3CA* and *CTNNB1* are rarely present in benign thyroid nodules and common in more advanced thyroid cancers (39). TERT mutations in particular have been associated with increased disease specific mortality, distant metastasis and radioactive iodine refractory disease (40,41,42). *BRAF^V600E^* mutations are associated with higher recurrence rates and mortality in thyroid cancer (43). However, it remains controversial whether mutational status provides further prognostic information to that already provided by more traditional prognostic factors such as patient characteristics and grade and stage of disease at presentation. For thyroid papillary microcarcinoma evidence suggested that *BRAF* status together with several histopathologic features was a better predictor of extrathyroid tumor spread than either mutation or histopathologic findings alone (44).

It is likely that with increasing application of mutational analysis in thyroid nodules and cancers, and analysis of prospective studies of its use will provide data to answer this question in the future.

It must also be taken into consideration that while factors inherent to the tumor, including mutation status, have effects on prognosis; “host factors” involving the patient harboring the tumor may independently, or via complex interactions with the genomic alterations of the tumor have effects on tumor behavior and prognosis. Underscoring this point is a recent report associating obesity with increased prevalence of *BRAF^V600E^* mutations among patients with PTC (45).

Targeted therapies directed by results of mutational analysis are recommended for a variety of cancers. Treatment of melanoma based on BRAF status and assessment of *KRAS* mutational status to determine medical treatment for colorectal cancer are strategies that have been tested in clinical trials (46,47). While targeted therapy based on mutational analysis is not yet a widely accepted practice for thyroid cancer there are several clinical trials in progress to test this hypothesis. Clinical trials of MEK or *BRAF* inhibitors to increase radioiodine uptake for patients with *BRAF*-mutant, RAI-refractory thyroid carcinoma (ClinicalTrials.gov Identifier: NCT02145143) and RAS mutated thyroid cancer (ClinicalTrials.gov Identifier: NCT02152995) are underway. Studies of combination of BRAF and MEK inhibitors for patients with BRAF mutant anaplastic thyroid cancer (ClinicalTrials.gov Identifier: NCT02034110) are also enrolling patients. Patients with advanced thyroid cancers harboring PAX8/PPARγ fusions (ClinicalTrials.gov Identifier: NCT01655719) and NTRK alterations (ClinicalTrials.gov Identifier: NCT02122913) are now being treated in clinical trials. Previous data showed that STRN-ALK fusions occur more often with aggressive types of thyroid cancer and several reports have demonstrated that patients with advanced thyroid cancer with ALK fusions may benefit from ALK inhibitors such as Crizotinib (48). Not previously seen in thyroid cancer, a mutation in the TSC2 and in the mTOR protein was discovered in a patient with metastatic anaplastic thyroid cancer who initially achieved a near-complete response to Everolimus with posterior resistance and progression of the disease. This shows the possible benefit of sequencing a patient’s cancer DNA prior to treatment and following disease recurrence, which may help guide treatment in patients with similar mutations (49).

## CONCLUSION

As a comprehensive genome atlas of thyroid cancer is rapidly becoming a reality, and with emerging methodologies such as NGS providing detailed genetic information regarding thyroid tumors, we have now entered into the genomic age of diagnosis and treatment of thyroid nodules and thyroid cancer. Several molecular tests are now available to assist in the diagnosis of thyroid nodules among which NGS appears to be a particularly promising tool that could most accurately characterize the genetic alterations underlying these neoplasms. ThyroSeq for targeted detection of mutation has been tested for its accuracy and performance in diagnosing malignancy among thyroid nodules with indeterminate cytopathology in two single-center studies and found to have both high NPV and an improved PPV when compared to existing molecular tests. Large-scale multi-center studies are needed to validate these preliminary findings. Furthermore, future studies are needed to determine the optimal use of NGS in clinical-decision making for patients with indeterminate cytopathology after thyroid FNA. Several studies and reports point to the potential impact that knowledge of mutational status of thyroid tumors can have on prognostication and selection of targeted-therapies, though it remains to be elucidated whether strategies to treat thyroid cancer based on mutational status will improve overall outcomes. As we look ahead to the era of “personalized medicine” NGS appears to hold promise as a potentially useful tool in the detection of thyroid malignancy as well as a possible aid for the clinician in determining optimal treatment for patients with thyroid neoplasia.

## Figures and Tables

**Table 1:**
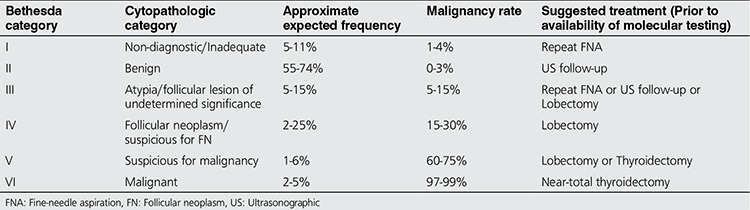
Bethesda system for the classification of thyroid cytopathology
